# Industrial Applications of Ionic Liquids

**DOI:** 10.3390/molecules25215207

**Published:** 2020-11-09

**Authors:** Adam J. Greer, Johan Jacquemin, Christopher Hardacre

**Affiliations:** 1 Department of Chemical Engineering and Analytical Science, the University of Manchester, the Mill, Sackville Street, Manchester M13 9PL, UK; 2 Laboratoire PCM2E, Université de Tours, Parc de Grandmont, 37200 Tours, France; 3 Materials Science and Nano-Engineering, Mohammed VI Polytechnic University, Lot 660-Hay Moulay Rachid, Ben Guerir 43150, Morocco

**Keywords:** ionic liquids, industrial applications, commercial processes, designer solvents, synthesis

## Abstract

Since their conception, ionic liquids (ILs) have been investigated for an extensive range of applications including in solvent chemistry, catalysis, and electrochemistry. This is due to their designation as designer solvents, whereby the physiochemical properties of an IL can be tuned for specific applications. This has led to significant research activity both by academia and industry from the 1990s, accelerating research in many fields and leading to the filing of numerous patents. However, while ILs have received great interest in the patent literature, only a limited number of processes are known to have been commercialised. This review aims to provide a perspective on the successful commercialisation of IL-based processes, to date, and the advantages and disadvantages associated with the use of ILs in industry.

## 1. Introduction

**Figure 1:**
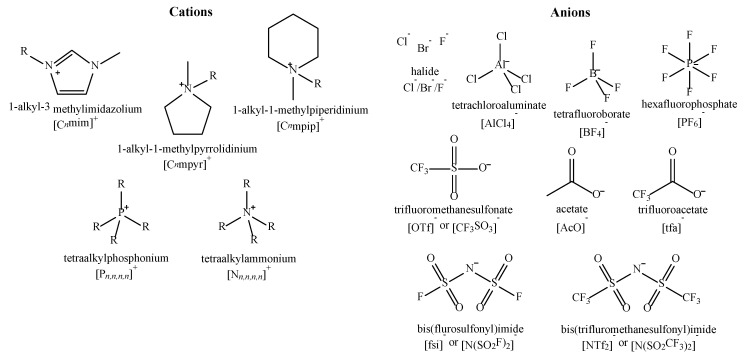
Examples of common cations and anions found in ionic liquids, and their preferred abbreviations.

Research in the field of ionic liquids (ILs) has been steadily increasing over the last two decades, since their initial discovery in 1914 by Paul Walden [1]. ILs are liquid salts comprised entirely of cations and anions and are frequently described as being liquid below an arbitrary value, such as 100 °C, or at room temperature (RT). It should be noted that this temperature constraint is not required for a substance to be considered an IL [2]. ILs are differentiated from other salts, such as sodium chloride, which only become molten at extremely high temperatures (>800 °C) due to their tightly packed structure and strong ionic interactions. The lower melting point of an IL is attributed to the bulky and unsymmetrical structure of the constituent ions, which can be paired together in different combinations to tailor their thermophysical properties [3]. Some examples of common cations and anions are shown in Figure 1, together with their preferred abbreviations [4].

ILs were branded as “solvents of the future” in 2003, due to the wide range of applications in which they could be used, particularly as replacements for traditional solvents in industrial processes which were often toxic, flammable and highly volatile [5]. Their low vapour pressure led to an assumption that ILs could also be considered as green solvents, due to low levels of atmospheric pollution, however, it is now well known that the manufacturing, use, and disposal of the solvent itself, must also be considered [6,7,8,9,10]. Furthermore, IL toxicity has been identified as an emerging problem as the use of ILs increases, particularly towards aquatic organisms, and studies have shown that this can be affected by changes in IL structure, such as the chosen cation/anion, alkyl chain length, and functionalised alkyl groups [11,12,13]. Some ILs containing fluorine anions, such as [PF_6_]^−^ or [BF_4_]^−^ are hydrolytically unstable and liberate HF, leading to an increase in popularity of the costly, but water stable, [NTf_2_]^−^ and tris(perfluoroalkyl)trifluorophosphate ([FAP]^−^) anions [14,15]. However, it is important to remember that in many cases, the solvent that the IL is replacing can be much more harmful (to both the user and the environment), such as carcinogenic chromium (IV) salts used in chromium electroplating processes, or HF used in alkylation reactions; yet nonetheless, detailed toxicity testing of proposed ILs is still essential.

Many ILs have a high thermal stability, and a low flammability owing to their negligible vapour pressure at ambient temperature. These properties can increase the safety of high temperature solvent applications, such as in batteries, or even allow their use in space [16]. It should be noted, however, that many ILs can still be distilled, but often extreme conditions are required [17]. Additionally, as ILs consist exclusively of ions, they also have higher thermal and electrical conductivities in comparison to common laboratory solvents, as well as larger electrochemical windows. This has meant that they have found uses as electrolytes and heat transfer fluids and can effectively disperse heat produced during a reaction.

**Figure 2:**
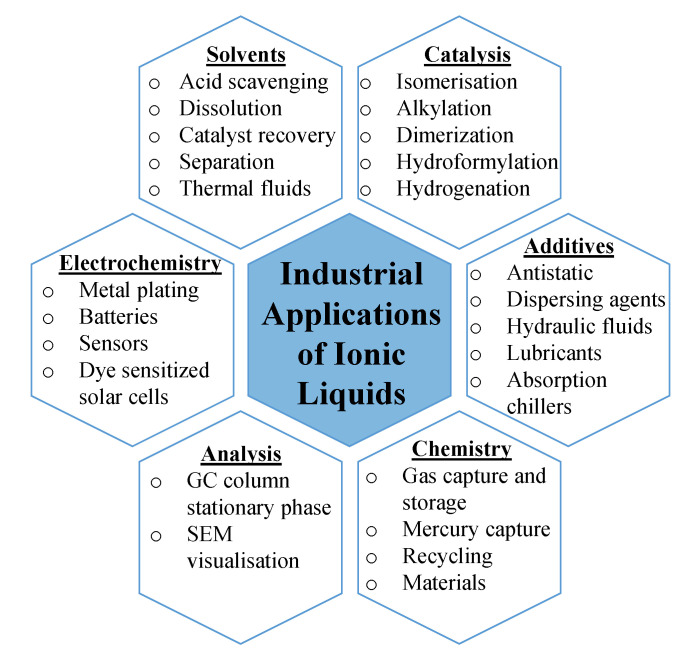
Illustration of some well-known applications of ionic liquids.

The idea that ILs could replace conventional solvents created a lot of interest in the academic and industrial communities. This substitution is enforced by the EU REACH (Registration, Evaluation, Authorisation and Restriction of Chemicals) regulations that are designed to improve safety and protect the environment, and their restricted substances list, which phases out hazardous chemicals and provides further opportunities for safer, IL-based processes [18]. However, prior to being able to substitute any organic solvent used in a given process, the compatibility of a selected IL must be checked in respect to all the operation units of a process. A comparison of the main properties of organic solvents vs. those of ILs originally reported by Plechkova and Seddon in 2008 has been updated herein, as reported in Table 1, to highlight the main differences between these two classes of materials [19]. The tuneable nature of ILs allows for the synthesis of a solvent with specific properties tailored to a particular application, which is less the case for organic solvents. In light of such a comparison, while ILs have uses as alternative solvents, many other applications exist as reported in Figure 2 [20,21].

In particular, ILs have been used in a number of industrial processes to improve catalyst recovery and product separation through the formation of biphasic systems. Their solvation properties are greatly influenced by the ions’ ability to act as hydrogen bond acceptors/donors, and the degree of delocalisation of the anionic charge [20]. They also possess a non-coordinating, highly polar nature, caused by weakly coordinating ions, meaning that ILs have been demonstrated to improve reaction rates and selectivity of numerous reactions [3,21,24,25]. While there have been numerous discoveries and patents filled in the field of ILs, the number which has reached commercial application is relatively limited. For example, the use of ILs as a drop-in replacement for conventional solvents in many processes has not been realised, often because other factors, such as increases in conversion/selectivity, ease of product separation, or process material compatibility [26], are desired in order for the process to be commercially viable, rather than simply considering the cost and safety/toxicity implications of the alternative solvent.

The earliest review article on the commercial prospects of ILs was published by Plechkova and Seddon in 2008, but there have been many recent advancements in the field since then [19]. Recent review articles did not address the scale of known industrial processes, until the recent publication of a book written on the *Commercial Applications of Ionic Liquids*, which gave a detailed insight into several industrial processes, and provided a list of 57 applications that have been commercialised or piloted utilising ILs [27]. The aim of this review article is to build on this and give a more detailed explanation of known IL-based processes, addressing why ILs were employed specifically in each application. Additional processes are also identified, herein.

## 2. Growing Interest from the Academic and Industrial Communities

**Figure 3:**
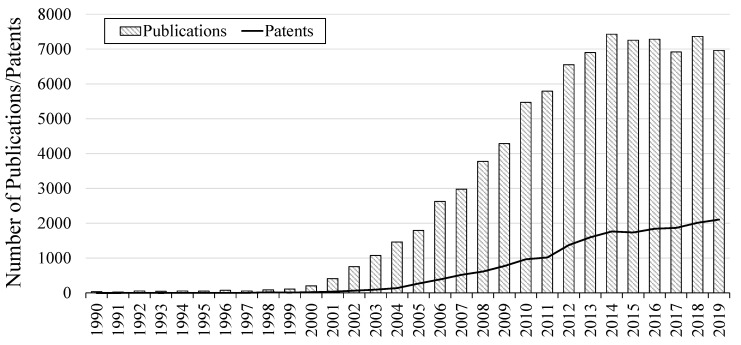
The number of publications and patents each year from 1990 to 2019, found in the SciFinder^TM^ database containing the concept “ionic liquid”. The addition of further keywords, such as “room temperature molten salt”, was found to be insignificant.

While haloaluminate ILs have been investigated since the 1950s, the first imidazolium-based IL was not studied until 1982 [28,29]. However, only with the development of “air and moisture stable” ILs were they seriously considered as viable alternatives to conventional organic solvents [30]. This was because haloaluminate-based ILs were very sensitive to moisture [31]. Welton reported that the 1990s marked the birth of a new field of IL research, where other academics were slowly becoming more aware of their existence [2]. Since 1990, there have been >87,000 publications in the field, however, data from 2014 onwards suggest a plateau in the year-on-year increase that has come to be expected (Figure 3).

**Figure 4:**
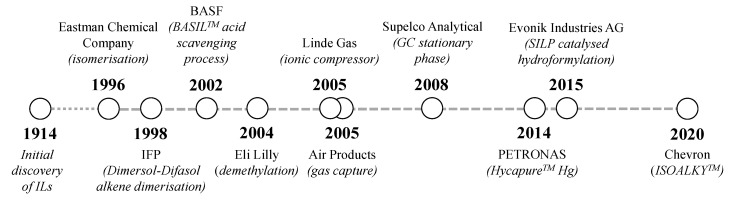
Development of commercialised IL-based processes.

In contrast, commercial interest in the widespread applications of ILs has continued to grow, demonstrated by the increasing number of patents displayed in Figure 4. As of 2019, more than 19,000 patents had been filed worldwide, with ~2100 published in 2019 alone. While the exponential growth has slowed in recent years, it demonstrates that there is now widespread awareness of the advantages of IL-assisted applications in industry. In 2018, Morton and Hamer from the intellectual property firm Mathys & Squire LLP, published a noteworthy article looking at the life of ILs through patent claims [32]. Interestingly, they found that between 2002 and 2015 there was a decrease in patents being filed with claims to the IL as a product (*per se*), and an increase in claims related to the application of ILs in a process (*use* or *process* claims). This is rational as the synthesis of ILs is now considered to be well described in the patent literature, whereas finding new uses for particular classes of ILs in specific applications is where there is room for growth. Although, as demonstrated by Proionic and their recently patented CBILS^®^ process, there is still space for further synthesis developments. The authors also noted that while “air and moisture” stable ILs were declared by the academic community in 1992, Exxon was granted a patent in 1982 describing the existence of such ILs with quaternary phosphonium/ammonium cations and, e.g., halide/[RSO_3_]^−^/[RCOO]^−^/[BF_4_]^−^/[CF_3_SO_3_]^−^ anions for the extraction of aromatics from hydrocarbon feeds [30,33]. Between 2001 and 2007, there was in increase in the disclosure of functionalised, or task-specific ILs, as well as the use of ILs in new applications [32]. This growth continued through to 2015, where Morton and Hamer identified that in many cases, it is simple, well-known imidazolium or pyridinium ILs that are being disclosed in recent patents, for use in new fields. Importantly, ILs are also being claimed generically in applications where the use of solvents is required, showing their prominence in many commercial areas.

## 3. Industrial Applications of ILs

Since the commercialisation of the first known IL-based process in 1996 (Figure 4), there has been an explosion in the use of ILs for a diverse range of applications. It is the aim of this section to detail many of the known processes that have operated on a commercial (Section 3.1) or pilot (Section 3.2) scale.

### 3.1. Commercial Scale Processes

The earliest reported example of the scale-up of IL technology was by the Texas Eastman Division of Eastman Chemical Company in 1996 for the isomerisation of 3,4-epoxybut-1-ene to 2,5-dihydrofuran, an intermediate in the manufacture of tetrahydrofuran [34]. The process required a Lewis acid catalyst and a Lewis base IL (tetraalkylphosphonium iodide) in a continuously fed reactor, and produced 1400 tonnes of product per year until 2004, when it was shut down as the market for the furan declined [35,36]. This was carried out in collaboration with Cytec Industries, a major IL manufacturer, that was acquired by Solvay in 2015 [37].

The first widely publicised example of a commercial process utilising ILs was the BASIL^TM^ (biphasic acid-scavenging utilising ionic liquids) process, announced by BASF in 2002 at their site in Ludwigshafen, Germany [38,39]. They successfully demonstrated that ILs could be handled on a large scale, and also effectively recycled, setting the scene for future industry applications [40]. The production of alkoxyphenylphosphine, a precursor to photoinitiators for inks and coatings, generates hydrochloric acid in the final stage of the reaction, which was originally scavenged through the addition of trialkylamine. The resulting ammonium salt had a significant disadvantage in that it formed a viscous slurry in the reactor, making separation of the desired product challenging. BASF discovered that when 1-methylimidazole was added instead, a protic IL (PIL) was produced, 1-methylimidazolium chloride, which was liquid above 75 °C, and resulted in the formation of two easily separated phases and an increase in reaction yield. The acid scavenging 1-methylimidazole could then be regenerated by adding sodium hydroxide. The reaction was scaled from a batch reactor to a continuous jet stream reactor in 2004, increasing productivity by a factor of 8 × 10^4^ to 690,000 kg·m^−3^·h^−1^, producing more than 1000 tonnes of product per year. This technology has a widespread applicability to any process that produces, or uses, acids.

#### 3.1.1. Electrochemical Applications

The inherent conductivity of ILs makes them suitable for a wide range of electrochemical applications, such as Scionix’s chromium electroplating process [34,41,42]. The system is based on choline chloride and chromium (III) chloride in the form of a deep eutectic solvent (DES), offering a safer alternative to toxic chromium (IV) salts, and avoiding embrittlement of the coating from the competing hydrogen evolution reaction observed when chromic acid-based electrolytes are used [43,44]. The use of ILs results in improved current efficiencies (>90%) and crack-free, corrosion resistant coatings. The IL technology is used in a variety of metal processing applications, including electroplating and electropolishing processes. Scionix’s founder (Abbott) has stated that there are two processes running on a commercial scale (>1 tonne), and seven further pilot scale processes (50–250 kg) for a range of different metals [45,46].

ILs have also found uses in batteries due to improvements in safety (non-flammable) and increased high-voltage stability. NOHMs Technologies commercialised ILs as an electrolyte (NanoLyte) in Li-ion batteries, yielding 400% more cycle-lifetime [47,48]; and NantEnergy (formerly Fluidic Energies) utilised ILs in Zn-air batteries [49,50,51,52]. Another electrochemical application exploiting ILs is dye sensitised solar cells (DSSCs), comprising a semiconducting IL-based electrolyte and photo-sensitised anode, with a dye that increases the cell’s sensitivity to visible light, and are a low-cost alternative to crystalline silicon cells. G24 Innovations Ltd., in combination with BASF, was the first known company to commercialise this technology, as early as 2008 [53]. Additionally, ILs have also been commercialised in electrochemical gas sensors (Novasina, IoLiTec) and supercapacitors (Panasonic) [54].

#### 3.1.2. Alkylation

Gasoline is manufactured from the alkylation of smaller hydrocarbons, such as C_3_-C_5_ olefins, produced during the catalytic cracking of crude oil in over 300 plants worldwide. Conventionally, the alkylation process is catalysed by a corrosive acid (H_2_SO_4_ or HF), however, it has recently been demonstrated that ILs are a suitable and safer alternative [55]. Chevron, in collaboration with QUILL (Queen’s University Ionic Liquid Laboratories), began investigating ILs as alkylation catalysts as early as 1999, and ran a demonstration unit with their ISOALKY^TM^ technology for 5 years from 2010 to 2015 to optimise reaction conditions [56,57,58]. The process utilises a chloroaluminate-based IL in combination with an organic chloride co-catalyst to produce a mixture with an extremely high acidity, but also a high hydrophilicity, requiring the reactant feed to be dried to <1 ppm to protect it from undergoing hydrolysis. Post-reaction, the IL is separated from the product stream using proprietary coalescing technology, diverting the product stream to a distillation column, and allowing recovery of the IL catalyst. In 2018, Chevron began retrofitting the IL technology to a refinery producing approximately 190 kton/year of alkylate, with the unit due to start up in 2020. The ISOALKY^TM^ unit, licensed by Honeywell UOP, requires a smaller amount of catalyst due to a higher activity, in comparison to the acid-based process, as well as significantly increasing product yield and quality while maintaining similar construction and operational costs. This is a significant development in the large-scale use of ILs for commercial applications [59,60,61].

The China University of Petroleum-Beijing have also commercialised an IL-based alkylation process, Ionikylation, comprising a mixture of trialkylammonium hydrochloride, CuCl, and AlCl_3_, licensed through Well Resources [57,62,63]. After an initial pilot in 2003, there are now six known units in operation, between 2013 and March 2020, collectively producing 1200 kton/year of alkylate, with another five units to be commissioned by the end of the year [64,65]. This has been required in order to produce large quantities of higher octane, low sulphur alkylate, due to increasing motor emission standards. It is likely that this is one of the largest commercial scale processes utilising ILs.

#### 3.1.3. Capture

Air Products (now Versum Materials, Inc.) reported in 2005 that they had developed a new method for storing and transporting toxic gases, e.g., PH_3_, BF_3_ and AsH_3_, which are widely used in the electronics industry to dope silicon [34,66]. To reduce the risk of toxic gases being stored under high pressure, these gases are conventionally stored in a cylinder at sub-atmospheric pressures, physically absorbed to a solid absorbent such as activated carbon or zeolites, and withdrawn under vacuum. Alternatively, it was found that ILs could be tuned to match the reactivity of the different gases through modification of the ion pair, with added benefits over porous solids of an enhanced heat transfer rate and the ability to be pumped. Lewis acidic ILs could reversibly complex with Lewis basic gases, e.g., PH_3_, in a 2:1 mol ratio (IL:PH_3_), and vice versa for Lewis acidic gases, e.g., BF_3_ and AsH_3_ [67]. A 1-alkyl-3-methylimidazolium tetrafluoroborate IL ([C_n_mim][BF_4_]) was chosen for commercial scale development with BF_3_ after showing long-term stability and good gas evolution rates with negligible loss in capacity of the sorbent after repeated cycles [66].

A collaboration between researchers in QUILL and PETRONAS led to the development of a novel method of removing mercury from natural gas streams, with an exceptionally short implementation period (<four years) [68,69,70,71,72]. The HycaPure^TM^ Hg process (licensed by Clariant in 2014) is based upon a chlorocuprate (II) IL impregnated on a high surface area support, or a so-called SILP (supported ionic liquid phase), combining the advantages of both homogeneous and heterogeneous systems. Mercury, which is largely in its elemental form in natural gas, is removed from the stream via oxidative dissolution, where the copper oxidises the mercury to form an anionic mercury complex, which is incorporated into the IL and produces a copper chloride by-product. Conventional methods of removing mercury vapour, such as chemically modified activated carbon, only have a lifetime a third of that of the IL and a lower efficiency, representing a significant cost saving and a reduction in mercury-contaminated waste. The technology was installed on a commercial natural gas plant in Malaysia, and after three years of continuous operation the mercury outlet concentration still remained low, meeting outlet specifications [68].

#### 3.1.4. Hydrogenation

Growing interest in SILP-based catalyst systems led to the investigation of scaled-up synthesis methods [73]. Conventional synthesis methods require the catalyst to be dissolved in an appropriate solvent followed by the addition of IL and a porous catalyst support material, and the subsequent removal of the solvent. The solvent should be removed slowly to ensure dispersion of the catalyst and IL across the porous support. Instead, it was shown that SILP materials could be reproducibly prepared using a fluidised-bed spray coating, whereby the support material is fluidised using a temperature controlled inert gas, and a solution of catalyst and IL in a solvent is subsequently sprayed onto the support. The temperature and pressure of the gas flow can be controlled, aiding evaporation of the solvent and ensuring a uniform dispersion and shell thickness, while post-treatment of the prepared SILP catalyst with solvent can tailor the catalytic functionalities. This process can also be used to synthesise solid catalysts with ionic liquid layer (SCILL), comprising a traditional catalyst coated with a thin layer of IL. Such a material was commercialised by Clariant for a hydrogenation process, where the IL layer affects the local concentration of reactants/products due to different solubilities, affecting the reactivity and selectivity of the reaction [54,74,75,76].

#### 3.1.5. Performance Additives

ILs have been exploited as performance additives by a number of industries seeking the benefit of their advantageous properties. Notably, 3M developed ILs as antistatic additives (at typically 1–5 wt.%) to improve cleanliness (reduce particles and dust) and also safety, by reducing electrostatic discharge events due to their high conductivity [77]. They can be designed to be lipophilic, ensuring high optical clarity and low haze, with good solubility in organic solvents, monomers, polymers and a low affinity for water (hydrophobic). Examples given of their products are quaternary ammonium salts with bis(trifluorosulfonyl)imide anions (3M^TM^ Ionic Liquid Antistat FC-4400). The ammonium cation can be tailored with a primary alcohol group to improve polymer compatibility, e.g., urethanes (3M^TM^ Ionic Liquid Antistat FC-5000). The ILs are compatible with a range of common processing methods (e.g., hot melt extrusion or solvent casting) and polymers (e.g., urethanes, epoxies, PVC, PVDF, PET, adhesives and coatings). BASF, Evonik and KOEI Chemical Co., Ltd. have also commercialised ILs for this purpose [78,79,80].

Evonik has explored the use of ILs as a secondary dispersant additive to homogeneously stabilise water-based pigments in water- and solvent-based paints and prevent sedimentation [81,82]. This employed ammonium- and quaternary heterocyclic-based ILs marketed under the Tego^®^ brand, and was a cost-effective universal solution for tinting paints to improve colour strength and brightness. The ability to design task-specific ILs was essential, as large R-groups could be specified to sterically stabilise the pigments or fillers used.

Furthermore, IoLiTec is known to manufacture and carry out its own research and development on IL-based performance additives for a wide variety of applications: dispersing agents (commercial), optical brighteners (commercial), cleaning additives (commercial), synthesis of inorganic materials with defined particle sizes (commercial), alcohol synthesis (pilot), high temperature protein stabilisers (pilot), and the purification of electronic display materials (pilot), however, information on these processes is sparse [54,83,84].

#### 3.1.6. Dissolution

Research funded by the U.S. Department of Defense led to the commercialisation of a “Natural Fibre Welding^®^” process [85,86]. In this process, ILs, such as [C_2_mim][AcO], have been applied to process natural fibres (cellulose, hemicellulose, silk, etc.) to form a congealed network which retains the native polymer structure [87]. A suitable amount of IL is introduced to the fibrous material which causes it to swell and solubilise the outer layers. This then becomes mobile and interacts with the surfaces of adjacent fibres, creating extended hydrogen-bonded networks, where the IL can then be removed using a solvent to create welded materials. The properties of the new material and the extent of the welding process can be controlled by conditions (time, temperature, pressure) or the IL (chemical nature, amount, placement). Natural Fibre Welding, Inc., has used this innovative technology to recycle (and even upcycle) existing natural materials to create new high-performance textiles for a variety of applications [88].

Plastic recycling is a problem also being tackled by IL-assisted technology. The difficulty posed by recycling plastics is that it is often more economical to incinerate or landfill the waste material. As of 2015, 6.3 billion tonnes of plastic waste had been generated, of which only 9% was recycled, with incineration (12%) and landfill (79%) accounting for the largest methods of disposal [89]. As well as this, plastics are often comprised of composite materials or contain additives, such as pigments or plasticisers, which require separation through mechanical or chemical processes to produce pure product streams, increasing costs [90]. However, there are certain plastics with chemical structures that lend themselves to easier chemical recycling; breaking the polymer back into monomeric forms for reprocessing. An example of this is PET (polyethylene terephthalate) which is widely used in food packaging, and it has been shown that this material can be degraded by ILs [91,92]. In comparison to mechanical recycling which reduces the quality of the product, chemical recycling offers the ability to break down polymers into monomers/raw products to be used as a feedstock to produce higher quality materials. Ioniqa, a spin-out company from Eindhoven University in the Netherlands, has developed a proprietary process for producing virgin quality plastic from waste PET, currently taking place on a 10,000 tonne per year scale [93]. The Ioniqa process, whose name suggests the use of ionic media, is detailed in recent patents which describe the formation of a high density dispersion of magnetic nanoparticles in a fluid [94]. A bridging moiety (e.g., weak organic acid) can be used to incorporate a magnetic nanoparticle and a “catalyst entity”, comprising a cation (e.g., [C_4_mim]^+^/[C_2_mim]^+^) and an anion (e.g., [FeCl_4_]^−^ or a halide), which is catalytically active towards the degradation of polymers into oligomers/monomers [95]. The tethering of a magnetic particle enables separation and recovery of the catalyst complex for reuse after the depolymerisation reaction. Furthermore, polymer additives such as pigments, or fillers, can be removed through this process [96]. The depolymerisation process is developed in partnership with Unilever, Indorama Ventures, and the Coca-Cola Company, and Ioniqa aims to use its 10,000 tonne plant as a basis to start selling licences worldwide for monomer production on the 50,000 tonne scale [97].

#### 3.1.7. Operating Fluids 

The use of ILs as operating fluids, rather than solvents, where they function as heat transfer materials or lubricants, is a well investigated area [98,99]. Mettop GmbH is a company specialising in technology for metallurgical processes [100]. In collaboration with Proionic, they developed a new cooling technology (ILTEC) to allow the direct substitution of water with an equivalent viscosity IL (IL-B2001) that could provide a higher operating temperature (<250 °C) and chemical inertness, in combination with energy savings through more efficient heat recovery [101,102]. It offered increased safety benefits in the case of a leak, where the IL would safely thermally decompose without a sudden increase in volume or production of hydrogen. IL-B2001 is comprised of [C_2_mim][BF_4_] (97%), [C_2_mim]F (1.5%) and water (0.5%) (1% unknown) [102]. Proionic uses a chloride free IL synthesis method, ensuring that the liquid is non-corrosive to the existing piping equipment (corrosion rate of 0.8 mm/year for copper/steel/Monel at temperatures up to 250 °C) [102]. The ILTEC technology consists of an IL filled storage tank that is purged with N_2_ to prevent the ingress of water, and two pumps (including one spare) to ensure the flow of IL through the cooling system. The secondary cooling circuit employs two heat exchangers. Industrial scale trials took place in 2012, where 35 L/min of IL was safely pumped into an anode furnace at a liquid copper temperature of 1200 °C with only slight bubbling of the melt [102]. In January 2015, the first industrial scale use of the technology took place in Norway, and since then there has been a minimum of three more operations in Europe [103,104].

Lubricants have been an important area of development for ILs due to their high thermal stability, conductivity, low volatility and low flammability. These properties are extremely important for high friction areas where there is the potential for heat to be generated, and the highly tuneable nature of ILs allows for tailoring of these properties, as well as viscosity, to specific applications. Klüber Lubrication commercialised the use of ILs to enhance the electrical conductivity of lubricants [105,106]. This was necessary due to increased levels of electric erosion as the use of electric motors grows, particularly in rolling bearings. Current is incidentally passed to the bearing which causes the localised generation of heat and subsequent pitting of the bearing surface. This eventually leads to premature failure of the bearing. Electro-erosion can be counteracted by insulating the inner or outer rings of the bearing; however, this is costly and often unsuccessful. Another method is to use “black” lubricants containing graphite or carbon particles, but such particles could hinder the smooth running of the bearing. Klüber found that ILs could be used to dissipate the generated current, and test rig data proved that damage arising from electric currents was reduced considerably. In a recent report, Proionic was also reported to have commercialised ILs as a lubricant in a high pressure gas compression process [54].

ILs were utilised as a liquid piston in an “ionic compressor” by Linde gas, as an alternative to conventional metal piston compressors [107]. The IL was designed to possess a low compressibility, and also a low miscibility with the compressed gas, allowing for simplification of the piston, reducing five hundred moving parts to eight; decreasing material and energy costs, as well as increasing servicing intervals by a factor of ten [108]. Furthermore, compressing gases to high pressures is extremely exothermic, requiring heat transfer to take place. Conventionally, heat exchangers outside the cylinder are used to remove excess heat, however, in this case, the high heat transfer capability of ILs could also be utilised to remove heat from directly within the cylinder, resulting in an isothermal process. This idea was initially conceived in 2002, and in 2005, the compressors were used to fuel a fleet of natural gas powered cars [108,109]. The technology is applicable for a variety of gases and in recent years, Linde Gas has supplied its equipment to more than 90 H_2_ refuelling stations worldwide, such as in Aberdeen, Scotland (2015), originally designed for refuelling single-deck buses [110,111]. 

#### 3.1.8. Analytical Uses

ILs have also found commercial uses in specialist analytical equipment such as stationary phases in gas chromatography (GC) columns and supports for scanning electron microscopy (SEM). Supelco^TM^ Analytical (a division of Sigma-Aldrich) introduced a range of GC columns with IL stationary phases in 2008 [112]. The IL-based columns had the advantage of being easily modified (polarity) with a greater thermal and chemical stability than commonly used stationary phases (polysiloxane polymers or polyethylene glycols). Di/polycationic systems (including imidazolium and phosphonium) were employed due to their higher thermal stability and increased separation efficiency, especially at higher temperatures [113]. Of note, 1,9-Di(3-vinylimidazolium)nonane bis(trifluorosulfonyl)imide (SLB-IL100) was the first commercially available IL-based GC column for the analysis of notably both neutral, and polarizable (containing C=C and/or C≡C bonds) analytes [112].

Researchers from Queen’s University Belfast have commercialised an IL-based separation process [114,115,116,117,118]. Countercurrent chromatography (CCC) is a continuous method of performing liquid–liquid separation on a commercial scale. The solutes are distributed between a stationary phase and a mobile phase consisting of two immiscible liquids, where one liquid is mixed and separated from the other phase in a rotating column/pipe. The stationary phase is held in place by hydrostatic and hydrodynamic forces while the mobile phase is pumped through it. While ILs have been commercialised in extraction processes due to their tuneable properties, limitations were found when using ILs in CCC and CPS (centrifugal partition chromatography) systems such as an increased backpressure due to the higher viscosity of the IL, however, later systems could be designed to exclude pressure bottlenecks. The developed system (ILPrep^TM^) was capable of being carefully designed by tuning of the IL viscosity and polarity to separate different transition metal salts, aromatics from aliphatics, or simple sugars (in collaboration with AECS-QuikPrep Ltd.) [119,120,121].

Hitachi High-Tech Corporation has developed an IL (HILEM^®^ IL 1000) to allow the visualisation of biological samples under high vacuum using SEM [122,123,124]. Importantly, the IL is hydrophilic in order to preserve the structure of hydrated specimens without dehydration/drying. While the negligible vapour pressure of ILs enables their use in low pressure environments, it was further discovered that an IL support could prevent the accumulation of electron charge, owing to its intrinsic conductivity. This could potentially replace the commonly used method of sputtering insulating samples with a thin layer of a highly conductive metal.

### 3.2. Pilot Scale Processes

#### 3.2.1. Electrochemical Applications

Many companies have been known to utilise the electrochemical properties of ILs on a pilot scale, such as C-Tech Innovation (aluminium plating), IoLiTec (aluminium plating), BASF (used 1-ethyl-3-methylimidazolium chloride for aluminium plating), and Xtalic (nanostructured aluminium alloy plating – XTALIUM^®^) [54,125,126,127,128,129]. The viscosity of the IL can hinder the mobility of the ions, and, therefore, the processes are often carried out at increased temperatures (up to 100 °C), although this is still lower than conventional processes [127]. Pionics Co. Ltd. developed ILs for use in lithium batteries and was building a mass-production plant for the technology in 2010 [130]. H.Glass and IoLiTec have piloted ILs in DSSC, supercapacitors (ZapGo), electrochromic windows (IoLiTec), and solvents/additives in redox flow electrolytes for Zn-Br batteries (IoLiTec) [54,131]. Degussa also observed that IL electrolytes could be integrated with lithium ion batteries to ultimately provide performance enhancements, however, there has been no evidence in the literature since that this has been commercialised [34].

#### 3.2.2. Demethylation

Eli Lilly and Company detailed an IL-assisted process in 2004 for the demethylation of 4-methoxyphenylbutyric acid to yield an important pharmaceutical intermediate [132]. A protic pyridinium hydrochloride IL was employed due to the harsh conditions of alternative methods (refluxing HBr in acetic acid), and it was found that the reaction was easily scaled up to operate on a 190 litre pilot plant. The high melting point of the IL meant that the reaction was carried out at temperature (>190 °C) for up to 5 h, and an excess of hydrochloric acid was required to ensure the product could be separated after the reaction by extraction with an organic solvent at room temperature (>94% yield). 

#### 3.2.3. Dimerisation

IFP (Institut Français du Pétrole) developed a homogeneous IL-based catalyst for the dimerisation of light alkenes. The Dimersol process was initially commercialised in 1977, utilising a soluble nickel salt and aluminium-based co-catalyst that was dissolved in a hydrocarbon and injected into a cascade reactor system with the alkene feed [133,134]. However, the catalyst was not easily separated from the reaction mixture and required removal through aqueous washing, resulting in lower yields and increased costs. This was later improved upon in 1998 with the biphasic Difasol process which solubilises the nickel-based catalyst in a slightly acidic imidazolium chloroaluminate IL [21,135]. This enhanced the separation due to a lower solubility of the dimerised products in the polar IL phase, compared to the monomeric reactants, therefore, increasing selectivity, yield, and catalyst recyclability. This technology could be retrofitted to existing Dimersol plants, or operated on its own, but so far has only operated on a pilot plant scale, potentially due to the capital cost of equipment. The Dimersol process was licenced (by Axens, an IFP subsidiary) to build thirty-five plants, processing more than 4.5 million tonnes of feed per year, showing the potential of this technology [133].

#### 3.2.4. Chlorination

The chlorination of hydrocarbons is commonly carried out using phosgene, however, this is a hazardous reagent with significant safety implications, and a less than ideal diol conversion. It is not widely known that BASF used ILs from 1990 in the form of low-melting Vilsmeier salts to activate HCl (stronger nucleophile) for the chlorination of diols on a pilot scale [66,134,136]. The resulting chlorinated product was immiscible with the salt, allowing simple separation and catalyst recycling.

#### 3.2.5. Hydrosilylation

Hydrosilylation is a reaction widely used to synthesise polyethersiloxanes for a range of uses such as emulsifiers, dispersants, or anti-foaming agents. These materials can be produced from a condensation reaction of alcohols with Si-Cl functionalities, however, this results in the production of HCl so, consequently, the metal catalysed reaction of alkenes with Si-H species is preferred [137]. It is catalysed by only a few ppm of a homogeneous Pt catalyst, so metal recovery is rarely carried out; however, this reduces the purity of the final product and would significantly increase the cost if removal was required [137,138]. Degussa, now part of Evonik Industries, focused on trying to recover the spent catalyst to improve purity by using a biphasic reaction mixture where the catalyst was dissolved in an IL. A range of imidazolium- and pyridinium-based ILs with [BF_4_]^−^/[RSO_4_]^−^/[NTf_2_]^−^ anions were investigated by Degussa for the process [137]. This reduced loss of the precious metal catalyst by improving phase separation, allowing for reuse of the IL-catalyst phase by decanting the pure product, and achieved conversions of >99% when operated on a pilot scale [139].

#### 3.2.6. Hydroformylation

Evonik Industries used a homogeneous IL catalyst in a hydroformylation reaction in 2015 [140]. Hydroformylation is the reaction of alkenes with CO/H_2_ to produce aldehydes using a metal catalyst (cobalt or rhodium), often in a biphasic aqueous system such as the industrial Ruhrchemie/Rhône-Poulenc oxo process, [141] or IL media, as shown by IFP Energy nouvelles (IFPEN) [21,142,143]. The process developed by Evonik makes use of a SILP catalyst, where early work demonstrated that it worked reliably for ~800 h with high selectivity; however, further optimisation was required to screen catalysts and the interaction of the bulky ligand with both the IL and reactant/product streams [140]. Ultimately, a rhodium complex with ligands based upon a polycyclic anthracene triol structure, dissolved in an IL consisting of an imidazolium cation and a binary amine anion, was tested on a pilot scale, where it displayed long-term stability of over 2000 h, bettering results obtained on the same system with no IL present. This was the first time a SILP system was considered attractive enough for commercialisation.

#### 3.2.7. Fluorination

Chlorofluorocarbons (CFCs), used predominantly as refrigerants, have been acknowledged to contribute to global warming and have been phased out in accordance with the Montreal Protocol [144]. CFCs were replaced by hydrofluorocarbons (HFCs) due to a lower ozone depletion potential, and are synthesised from the reaction of chlorohydrocarbons with HF; however, the antimony pentachloride catalyst commonly used suffered from deactivation and excess chlorine gas was required to re-oxidise the metal [145]. In 2001, Arkema filed a patent teaching the use of an IL with a chloro- or fluoro- antimony-based anion to catalyse the fluorination reaction [146]. The IL could be used to circumvent the catalyst deactivation, and Arkema demonstrated that the fluorination reaction could run for >1000 h with a selectivity higher than 99.5% on a micropilot scale [145].

#### 3.2.8. Water-Gas Shift

SILPs have also been studied by Clariant in the water-gas shift reaction (WGSR), a process for producing CO_2_ and H_2_ from CO and H_2_O. Clariant tested a Ru-SILP catalyst in an industrial WGSR plant using bypass tubing to hold 1.5 kg of the catalyst, acting as a fixed-bed reactor [147]. The pilot scale reaction demonstrated complete conversion of a feed containing ~20% CO, although a large exotherm of the reaction resulted in thermal decomposition and deactivation of the catalyst.

#### 3.2.9. Extraction

Rare earth elements are often found together in the Earth’s crust and are exceptionally difficult to separate from one another due to a similar chemical structure. They are necessary for numerous catalytic, electrical, magnetic and optical applications. Industrial separation methods focus on solvent extraction due to its recyclability and scalability using organophosphorus derivatives, however, this produces vast amounts of acidic waste and requires a large number of stages [148]. Increased demand for these materials is leading to supply problems, pushing researchers down the path of recycling used devices to recover these elements (urban mining). The growth of electric vehicles has further exemplified this problem, where it is estimated that 1–2 kg of rare earth metals is required for the permanent magnet motor in each vehicle. Recently, Queens University Belfast and Seren Technologies have filled several patents in the use of task-specific ILs to extract and separate rare earth metals found in permanent magnets (namely, neodymium and dysprosium) from an acidic aqueous solution [149,150,151]. They reveal that the metals can then be selectively removed from the IL phase through precipitation or electrodeposition. Using this knowledge, Seren Technologies opened a pre-commercial magnet recycling plant in England in 2018, offering a highly selective process that is capable of receiving multi-ton shipments of magnets, and is actively seeking commercialisation [152]. 

#### 3.2.10. Separation

The hygroscopic nature of ILs facilitates the breaking of the azeotropic interaction by entraining water, allowing for distillation of the other component [153,154,155]. BASF ran such a process on a pilot plant for three months, using 1-ethyl-3-methylimidazolium tosylate, where the low volatility and high thermal stability of the IL proved paramount to the recyclability of the system [156]. Eastman Chemical Company investigated ILs in an extraction process, using ILs with a quaternary phosphonium cation and a phosphinate or carboxylate anion to extract carboxylic acids from aqueous streams [157,158,159]. IoLiTec considered ILs in a CO_2_ capture process, which has been an area of significant interest in the literature [54].

#### 3.2.11. Dissolution

Due to the tuneable solvent properties of ILs, they are well suited for applications related to the selective dissolution of materials that are insoluble in water or other common laboratory solvents. ILs can add further benefits to a process due to a lower environmental impact in comparison to volatile chemicals and increased stability. For example, cellulose, found in the walls of plant cells, can be dissolved and reshaped/processed into fibres by the non-derivative Lyocell process using *N*-methylmorpholine-*N*-oxide (NMMO), however, NMMO still suffers from a low thermal stability [160]. In comparison, the viscose process utilises the toxic solvent, carbon disulphide. 

In 2002, Rogers investigated the dissolution of cellulose using imidazolium based ILs with Cl^−^, Br^−^, [BF_4_]^−^, [PF_6_]^−^ or [SCN]^−^ anions, finding that [C_4_mim]Cl could dissolve up to 25 wt.% cellulose aided by microwave heating [161]. They proposed that anions that were strong hydrogen bond acceptors were more effective, as they were able to solubilise cellulose by hydrogen bonding with the functional hydroxide groups. Extrusion of the IL-cellulose solution into water caused the cellulose to precipitate out and allowed for the preparation of thin fibres/rods, with important applications in the fibre, polymer, membrane and paper industries. The technology was patented and later licenced exclusively by BASF in 2005, who carried out further investigations into cellulose dissolution/re-shaping using [C_2_mim][AcO]. They later found that carboxylate-based ILs adversely reacted with cellulose through the formation of *N*-heterocyclic carbenes, and noted that chloride ILs can cause hydrolytic cleavage and degradation of cellulose when the water content increases above a certain threshold, which could be controlled by keeping the temperature above 100 °C [162,163,164]. Nonetheless, this promising work led to the widespread investigation of using ILs to dissolve biomass, as can be seen from the additional examples below.

An alternative Lyocell-type process utilising ILs is the closed-loop Ioncell^®^ process, developed by Aalto University in Finland for the dissolution of wood pulp, textiles and even newspapers [165]. Therein, it was demonstrated that superbase ILs, such as 1,5-diazabicyclo[4.3.0]non-5-ene acetate ([DBNH][AcO]), displayed better performance than alternative “first-generation” ILs that were shown to induce cellulose degradation as previously described [166]. The Ioncell^®^ process can be used to solubilise pulp, before dry-jet wet spinning the solution to produce highly orientated cellulose fibres with a higher tenacity than commercial viscose and NMMO-based Lyocell fibres, and the further advantage of a viable solvent recovery step. Aalto is constructing a pilot plant capable of producing 10 kg of fibre per day that is due to open this year, and anticipate a 5-year development phase before reaching commercial scale production [55].

Metsä Spring, a corporate venturing arm of the Metsä Group set up in 2018, is developing a new textile fibre production concept utilising an IL-based wood pulp dissolution process [167,168]. In 2018, over 100 million tonnes of textile fibres (e.g., polyester/nylon/cotton/hemp/cellulose) were produced, however, these are not considered environmentally friendly due to their production methods (viscose, or oil refining). The use of innovative solvents (such as ILs) and closed-loop manufacturing systems means that cellulose-based textile fabrics are becoming more sustainable. Metsä Spring is currently building a new test plant, budgeted at EUR 40 million, with the capacity to produce 40 kg of fibre per hour [167].

The pre-treatment of lignocellulosic biomass to separate cellulose from hemicellulose and the organic polymer, lignin, has being investigated using PILs in the ionoSolv process, developed at Imperial College London [169,170,171]. The high cost of the IL solvent had thus far prevented the widespread application of this technology on an industrial scale, however, the use of inexpensive PILs, such as triethylammonium hydrogensulfate ([N_2220_][HSO_4_]), was found to significantly reduce the bulk price of the solvent to an estimated USD 1.24/kg [172]. The water tolerance of the process is a further key benefit, avoiding the anhydrous conditions normally required. In the ionoSolv process, cellulose is selectively extracted from biomass by dissolving lignin and hemicellulose in a heated aqueous IL solution [171]. The highly delignified cellulose can be recovered and enzymatically hydrolysed into glucose for the production of biofuel. The lignin is precipitated from the IL solution by the further addition of water, while the hemicellulose can be pre-extracted and isolated as furfural or fermented as a separate sugar stream [169]. The pre-separation of lignin using this process reduces the capital cost of pre-treatment during the production of glucose by 30%. Lixea (formerly Chrysalix Technologies), a spin out company from Imperial College London, is commercialising ionoSolv fractionation technology in their BioFlex process [173,174]. The pre-treatment process offers feedstock flexibility, to include different types of biomass such as waste wood, forestry and agricultural residues, or sustainably grown biomass. The BioFlex process can even cope with heavy metal contamination (copper, chromium or arsenic), often used as preservatives, and these can be recovered from the IL via electrodeposition [175]. The process has operated in two 200 L scale-up tests at the Bio Base Europe pilot plant, with plans to design and build a pilot plant (ca. 200 tonnes per year) [176].

While cellulose is one of the most abundant polymers on earth, chitin, found in the hard shells of crustaceans, is the most abundant polymer in the marine environment [177]. Chitin is a linear amino polysaccharide, with a similar structure to cellulose where one hydroxyl group in the monomer is replaced by an acetylamine group, and has uses as a biopolymer in biodegradable packaging materials, controlled drug release formulations, food preservation or cosmetics, due to its bioactivity/compatibility and low toxicity [178]. Conventionally, chitin is produced by acid washing (demineralisation) the crushed shells, followed by an alkali wash to remove proteins and a decolourisation step. Due to a vast network of hydrogen bonds, chitin is entirely insoluble in water and most organic solvents, however, Rogers et al. discovered that basic 1-ethyl-3-methylimidazolium acetate ([C_2_mim][AcO]) could selectively dissolve the chitin, with microwave heating improving the dissolution efficiency [178]. Water could then be used to precipitate the chitin and subsequently dried to achieve a 94% recovery, with a higher purity and molecular weight than that achieved by the commercial process. The increased molecular weight allows the recovered chitin to be spun into fibres and used for a wide variety of applications [179]. From a 100 mL scale using a domestic microwave in 2010, to a 3 L pilot study with a continuous flow 2 kW microwave funded in 2012, Rogers was eventually able to move onto a larger 20 L pilot to conduct further scale-up and cost analyses [180]. This work resulted in Rogers cofounding Mari Signum, Mid Atlantic, LLC, a company interested in the production of high quality chitin and chitin-based products, who licenced the IL technology in 2016 [181]. Mari Signum is currently building a commercial processing plant, with an estimate that the plant capacity could eventually produce 90 tonnes of chitin annually [182].

#### 3.2.12. Operating Fluids

Evonik investigated ILs for application in absorption chillers as a replacement for commonly used aqueous LiBr [183,184]. ILs offered an advantage due to a wider liquidus range and increased corrosion protection at high temperatures, while maintaining a similar performance coefficient to LiBr systems. The flexibility in operating parameters of such a system would have a favourable impact on costs, and Evonik had plans to launch this technology in 2012 after field trials were completed. Evonik has also developed ILs for use as humidity control agents in liquid-based humidity-controlled air conditioning, with Chubu Electric Power Co., Inc. and Dyna-Air Co., Ltd. [185]. The use of non-corrosive ILs has the potential to reduce manufacturing cost of the units by more than 20% through enabling the use of cheaper metals (iron/steel/aluminium) in comparison to the corrosion resistant titanium required for LiCl solutions. Furthermore, less IL is required, dropping the power consumption of the circulation pump by 80%. Dyna-Air aimed to commercialise the product in 2019, however, no further information was available at this time.

## 4. Industrial Synthesis of Ionic Liquids

It can be seen from the previous section that ILs are currently used and investigated for an extensive variety of applications in bulk solvent (dissolution/extraction/separation), catalytic (hydroformylation/alkylation), additive (anti-statics/dispersing agents), and electrochemical (electrolytes for batteries/electrodeposition) technologies. Even more, this ranges from well-recognised processes such as BASF’s acid scavenging reaction, to more niche dissolution and additive applications. This shows that IL-based routes can compete on the same platform in terms of both cost and performance, in comparison with alternative technologies. However, the commercial applications of ILs can only be realised if sustainable and scalable synthesis procedures exist. Often, the ILs that have been commercialised in industrial scale processes are based upon simple, well-known salts, such as tetraalkylphosphonium iodide in Eastman Chemical Company’s isomerisation process. In speciality chemical applications, more complex systems have been developed using task-specific ILs, and this is often the focus of academic research. This has a clear effect on cost, where more synthetic steps are required, increasing the price of the IL. In some scenarios, the availability of starting materials can limit the scaled-up production of an IL, however, many common anions are available in large quantities in the form of metal salts or acids. The accessibility to large amounts of key reactants is an important identifier that a particular IL may be suitable for synthesising on a commercial scale.

Over the last three decades, the production of ILs on an industrial scale has increased. Collectively, this augmented the ability to investigate and facilitate IL-assisted processes, at scale. Many companies now offer ILs as part of their existing chemical portfolio, e.g., Acros Organics, BASF (Basionics^TM^), [186] Cytec Industries (part of the Solvay Group), [187,188] Evonik Industries, Merck, TCI, SACHEM and DuPont, while others are bespoke IL manufacturers, e.g., Iolitec, Proionic, Scionix and Solvionic (Table 2). Many of these companies are also involved in their own research and development, such as IoLiTec, as demonstrated in the previous sections. It is now estimated that up to 500 ILs are commercially available on the 1 g to 10 kg scale, dozens available on the 10 to 1000 kg scale, and 10 ILs available in greater than 1 tonne quantities [55].

**Figure 5:**
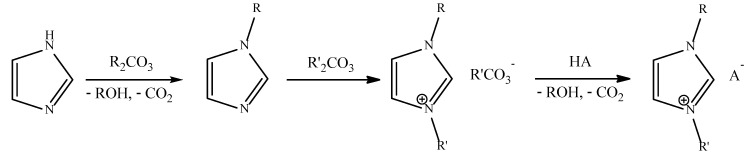
The CBILS^®^ (Carbonate-Based Ionic Liquid Synthesis) route for a 1,3-dialkylimidazolium based IL [196].

Proionic recently developed a novel IL synthesis method currently operating on the tonne scale [193,194,195]. The CBILS^®^ (Carbonate-Based Ionic Liquid Synthesis) route is completely halide free (<5 ppm), and can be used to produce pure imidazolium, pyrrolidinium, and phosphonium-based ILs (amongst others) [196]. Figure 5 shows an example of the synthesis of an imidazolium-based IL. The desired 1-alkylimidazole is synthesised by alkylating 1*H*-imidazole or a tertiary phosphine/amine with a carbonic acid dialkylester (R_2_CO_3_) at temperatures up to 130 °C. The 1-alkylimidazole is then reacted with a further R’_2_CO_3_ species in an alcohol solvent (R’OH) at 100 to 150 °C, to produce 1,3-dialkylimidazolium alkylcarbonate. The final step introduces the chosen anion through titration with any Brønsted acid (HA) with a pK_a_ lower than nine, hydrolysing the alkylcarbonate anion and producing the final product, which can be purified by simple evaporation. The alcohol and CO_2_ by-products can be recycled back to R_2_CO_3_, ensuring the process is also waste free. It has also been shown that this process can operate under continuous flow [197]. Continuous flow techniques present a more sustainable method of IL synthesis, with improved heat/mass transfer, and process intensification, as it can be operated uninterrupted for extended periods of time [36].

Solvionic specialises in manufacturing high purity ILs for catalysis, surface preparation and energy storage applications [198]. Solvionic stated that “The applications of IL based electrolytes are entering into the industrial phase for electrochemical storage systems. Solvionic, being a leader in the production and development of ILs and electrolytes in this field, is investing in innovative production lines to cater to this growing market demand. Annual production volume is targeted at 1.5 T/month by 2021, followed by the building of a few 4 T/month production lines by 2023 to achieve 50 T/month. This innovation will see a decrease in the market price of the ILs by 10 times” [189].

A widely acknowledged disadvantage of ILs is their respective cost, however, an IL-assisted process can bring with it further benefits, such as improvements to catalyst/solvent recyclability, increased product separation, and faster reaction rates, offering economic benefits in other areas. A commonly discussed example is the [NTf_2_]^−^ anion, used in many electrochemical applications, whose synthesis entails a costly 37 steps [55]. The large number of steps increases energy consumption and waste, decreasing the “greenness” of the material. This demonstrates that it is not always possible to reduce the cost of certain ILs, and that cheaper, easier to manufacture, replacement ions should be sought out with similar properties. Conversely, PILs offer a cheaper alternative, however, drawbacks such as incomplete ion exchange must be considered, and require measurements of ionicity [199].

Additionally, the use of ILs can affect the overall economics by improving reaction rates and producing higher quality products that are more easily separated from reaction mixtures, showing that the initial cost of the IL is not the only important factor. The use of biphasic systems improves the separation of the catalyst/products and aids complete recovery of expensive precious metal-based catalysts. The cost can also be offset by associated increases in safety from the replacement of toxic and volatile solvents. The higher initial cost of ILs is certainly not prohibitive, if judged by the number of commercialised processes. Furthermore, the development of continuous synthesis processes is hoped to drop the cost of IL production further. The CBILS^®^ process by Proionic is an example of this.

Some examples of the economy of scale for aprotic ILs can currently be found on the Proionic website [200]. [C_2_mim][NTf_2_] is available for EUR 690 kg^−1^ on a 1-kg scale, dropping to EUR 400 kg^−1^ for 150 kg. Recently in the literature, the [fsi]^−^ anion has been receiving increased interest as an alternative to the [NTf_2_]^−^ anion in electrochemical applications, due to a lower viscosity and higher conductivity [201]. [C_2_mim][fsi] can be purchased from Proionic for EUR 800 kg^−1^ on a 1 kg scale, but decreases by a factor of 2.3 to a lower price of EUR 350 kg^−1^ when considering a 150 kg scale, in comparison to a factor of 1.7 for [C_2_mim][NTf_2_]. Proionic also offer several [C_2_mim]^+^-based ILs with methanesulfonate, acetate and trifluoromethanesulfonate anions on a 150-kg scale, priced at EUR 135 kg^−1^, EUR 170 kg^−1^ and EUR 215 kg^−1^, respectively. In the case of PILs, such as those used in Lixea’s BioFlex biomass fractionation process, a recent scale-up study has shown that these can cost as little as USD 1.24–5.88 kg^−1^, with the implication that they are now in the same bracket as conventional organic solvents (USD 1.30–1.40 kg^−1^) [172]. Both these examples show that the increased demand for ILs is now achieving lower production costs and it is likely that these costs will decrease further (to a certain extent) as the quantity continues to go up.

## 5. Outlook

**Figure 6:**
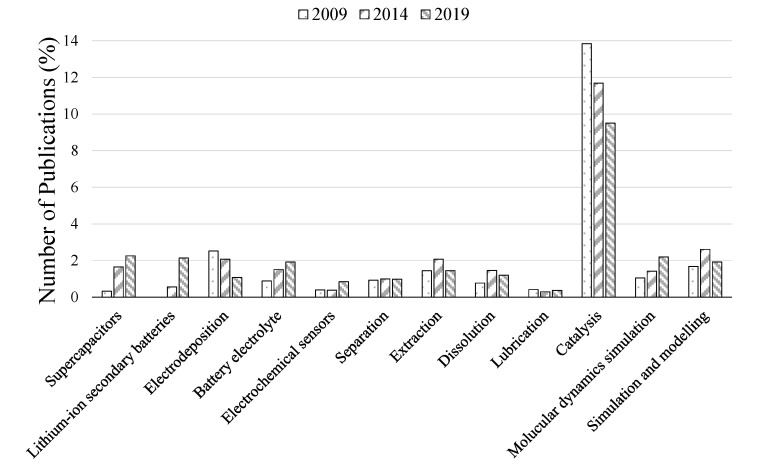
The number of ionic liquid publications in 2009, 2014 and 2019, over a selection of topics found using the “Categorize” function in SciFinder^TM^. The data have been normalised by the total number of publications in that particular year.

The increasing number of areas in which ILs are being employed can be demonstrated through academic publications, giving some direction as to the future of commercialised IL applications. Figure 6 displays a selection of different applications of ILs and compares how the number of publications in each area has changed between 2009 and 2019. For example, if the electrochemical applications of ILs are considered, there has been a shift away from electrodeposition and towards lithium-ion batteries. This suggests that ILs are being used more as speciality chemicals where cost is not a prohibiting factor, rather than bulk solvents/electrolytes where they have to compete with the lower price of organic solvents. This agrees with the increases found for supercapacitors and electrochemical sensors. Interest in other bulk solvent areas, such as separation, extraction and lubrication has remained similar; however, an increase in dissolution papers was found. This is further compounded by the number of recently commercialised dissolution processes.

Surprisingly, the number of catalysis related publications has been decreasing over the past decade. A closer look at the data, however, suggests that this in in fact due to an increasing number of areas in which ILs are being studied, with 593 publications in 2009 compared with 662 in 2019. This differs with changes in other fields where there are clear differences in the number of publications over the last decade, such as the lithium-ion secondary batteries category (as defined by SciFinder^TM^). It should be noted that while this observation may mean that while there has been a stabilisation in the number of publications over the last six years (Figure 3), the number of research topics is still increasing, bringing the use of ILs to more applications.

Furthermore, although simulation and modelling have long been used to predict IL properties and optimise the combination of ions, QSAR (quantitative structure–activity relationship) and QSPR (quantitative structure–property relationship)-based models now seem to be widely used due to the net improvement of the artificial intelligence [202,203,204]. Due to the many possible combinations (>×10^6^) of cations and anions, questions are raised regarding how to identify the optimum ion pairing for a specific application. However, thanks to the intensive free-to-access database, ILThermo, generated and updated by the National Institute of Standards and Technology (NIST) [22,23], several research groups have successfully developed predictive tools to evaluate or predict some properties of pure ILs (density, viscosity, thermal and electrical conductivity, speed of sound, heat capacity, etc.) [205,206] as well as in mixtures with other fluids (phase equilibria, thermodynamic and transport properties of IL-based mixtures, etc.) [207,208,209,210,211,212] through using group contribution models [213,214,215,216,217,218,219,220,221,222,223,224], QSPR [225], empirical or semi-empirical equations [226,227,228], ab-initio [229,230], molecular dynamic simulations [231,232,233], as well as equations of state [234,235,236].

Prior to being able to truly estimate their properties in silico, each collected dataset must first be critically evaluated to provide a unique recommended dataset for a given property of an individual IL as a function of temperature and pressure. The critical evaluation of the data is generally performed by using statistical and non-statistical analysis of collected datasets [237]; however, during this analysis the uncertainty in a given property caused by the purity (i.e., presence of impurities like water, halide and/or metal) of the IL must also be taken into account to avoid any false results or conclusions, as reported elsewhere [238]. The accurate reporting of physical properties is essential when considering the use of ILs in scaling-up models. Unfortunately, a worrying amount of literature is lacking essential physical characterisation, and most significantly the halide, metal and water content of synthesised ILs. It is well known that the presence of unreacted starting material, halide and metal impurities, or water can have a significant effect on the physiochemical properties (e.g., viscosity, melting point, thermal stability, etc.) of the final IL and negatively affect metal catalysed reactions [239,240]. This is especially important on larger scales, where conventional purification methods are not always scalable, and can significantly affect the cost. Thomas Schubert (IoLiTec) recently made a number of comments regarding the purity of ILs, where he found that the level of purity desired by customers sometimes led to adverse side-effects, where an IL that was thought to be a liquid at room temperature was delivered as a solid [241]. This was due to advanced purification procedures to remove unwanted impurities, resulting in an IL with a higher than expected melting point.

A similar rationale also applies to thermal stability, as when considering an IL for a process operated at elevated temperatures, it is arguably a key requirement that the measurement is carried out isothermally at the process operating temperature, and under the correct atmosphere (e.g., air or nitrogen) [205]. Furthermore, this can apply to the measurement of all temperature dependant transport properties including thermal conductivity, heat capacity and viscosity, to accurately determine economic costings. With these conditions of analysis, recommended datasets can then be used to implement fitting parameters of established equations (like the DIPPR 105 which describes the density of a fluid as a function of temperature) into chemical engineering software such as Aspen Plus, to properly describe ILs prior to being used in any chemical-engineering process and cost evaluations. In addition to the development of models for evaluating the physical properties of ILs, there is also a need for accurate datasets to further allow the construction of life cycle analysis (LCA), life cycle costing (LCC), techno-economic assessments (TEA) models to aid the scale-up process and allow researchers to focus on economically sustainable processes [242].

It should be realised that the chosen ions can be limited by commercial availability and cost, and so, a compromise must be found. It should also be noted that while academic researchers have the freedom to design complex IL systems, a large drawback to the commercial use of these solvents is their REACH status (in Europe). Finally, it should be observed that the complexity of the IL structure, and level of purity, should only be as high as is necessary for a particular application [243].

The continuously increasing number of areas in which ILs are being employed demonstrates the potential for future applications. Many of these applications are surrounding the use of ILs in electrochemical devices and energy storage. Novel polymerised ILs (polyILs) combine the high ionic conductivity and electrochemical stability benefits of the IL, with the mechanical stability and flexibility of polymers [244]. PolyILs can find uses in solid-state batteries, offering a leak-free, non-volatile, and non-flammable alternative to classical liquid-based electrolytes [245]. Furthermore, the amphiphilic nature of many ILs can induce self-assembly of highly ordered nanostructures that enhance charge storage [246]. The development of advanced IL-based materials presents an opportunity for increased ion conductivities for many electrochemical applications [247,248]; however, to the best of our knowledge, the synthesis of these materials is not yet carried out on an industrial scale, presenting problems with their commercialisation. As demand for new battery materials increases, developments in the synthesis of these materials may speed up in response. These improvements are further forced by regulatory changes, such as climate change (decreasing CO_2_ emissions) and the increasing use of batteries (e.g., electric vehicles).

On the topic of climate change, there has been a clear shift away from CCS (carbon capture and storage) and towards CCU (carbon capture and utilisation). While ILs have been widely investigated for the capture of CO_2_ and other acidic gases due to their tuneable absorption enthalpies and low vapour pressures [249,250,251,252], the chemical absorption of gases can also open up new possibilities for CO_2_ conversion into useful products and fuels through lowered activation energies [253,254,255]. Following on from this, IL encapsulation is another area of increasing interest for gas absorption and many other applications, due to the increasing surface area of the IL while maintaining bulk properties, in order to optimise activity [256]. The tuneable properties of ILs allow their use in niche applications where the often more complicated structures provide increased performance, while the associated higher cost is not perceived as a significant barrier in terms of speciality chemicals.

## 6. Conclusions

ILs have not been used in commercialised processes solely due to their “green” properties, but that is not to say that properties, such as a low volatility or low flammability, have not contributed to the overall success. The fact that ILs are comprised entirely of ions has led to their commercialisation in applications where high electrochemical and thermal stabilities/conductivities are required—properties unequalled by other pure materials. They are typically more expensive than conventional solvents, however, the initial increase in capital cost can be offset by improvements in solvent recyclability, catalyst recovery, reaction rates, selectivity, and product separation, as has been shown by a number of processes described within. Their use in industry is not confined to only bulk solvent applications and catalysis, with many niche processes now arising in areas of performance additives and analytical materials. Strong solvation properties and their tuneable nature can offer significant advantages in comparison to conventional solvents, however, the drop-in replacement of ILs in a process does not often occur without further consideration of process compatibility.

While ILs are not “inherently green”, they can improve the green metrics of a process by making it more sustainable, both environmentally and economically, with increased recyclability of the solvent and recovery of the catalyst. Tailoring of the hydrophilicity of the IL can be advantageous in terms of product separation, however, the effect of structural changes on the toxicity of the IL should not be forgotten. Furthermore, it should be noted that the price of ILs is now consistently decreasing with the development of novel and scalable synthesis routes, and some PILs are now estimated to be a similar price to conventional organic solvents, opening up the further exploration of these materials in industrial applications. It is estimated that further growth in the commercialisation of IL-based applications will be observed over the next decade, and that the topic areas will continue to grow as ILs are further accepted as generic solvents that should be considered when trialling any reaction. Additionally, attention should be given to alternative classes of solvents such as switchable solvents [257,258], and porous liquids [259,260,261], which have the possibility to provide benefits to the same types of processes in the future.

This review has demonstrated that close collaboration between academia and industry has been key to the success of many of the identified processes, and many university spin-out companies have also been generated. The cooperation enables academic research to progress in directions that are relevant to commercial applications, by aligning research aims to meet strategic targets such as performance improvements or cost. The commercialisation of IL-based processes is still at an early stage, and it is expected that there are still many exciting opportunities that are yet to be realised.

## Figures and Tables

**Table 1:** Comparison of organic solvents with ionic liquids (ILs), updated from work by Plechkova and Seddon [19]. Range of values were analysed from the NIST ILThermo database at 25 °C and atmospheric pressure [22,23].

Property	Organic Solvents	Ionic Liquids
Number of solvents	>1000	>10^6^
Applicability in a given process	Single function	Multifunction
Cost	Generally cheap	2 to 100 times more expensive than organic solvents
Recyclability/Toxicity	Green imperative—survey of toxicity of organic solvents is controlled by REACH	Economic imperative—toxicity and biodegradability are often not well known
Vapour pressure	Measurable and generally well-known—several organic solvents have vapour pressure > limit used in the classification of volatile organic compounds (VOCs)	For aprotic ILs: negligible vapour pressure under normal conditions
Flammability	Usually flammable	Usually non-flammable, but some ILs are used as propellants
Tuneability	Limited range of solvents available	Virtually unlimited range means “designer solvents”
Chirality	Rare	Common and tuneable
Catalytic ability	Rare	Common and tuneable
Viscosity/mPa·s	0.2–100	20–97,000
Density/g·cm^−3^	0.6–1.7	0.8–3.3
Refractive Index	1.3–1.6	1.3–2.2
Electrical conductivity/mS·cm^−1^	Usually insulator	Up to 120
Thermal conductivity/W·m^−1^·K^−1^	0.1–0.6	0.1–0.3

**Table 2:** Bespoke manufacturers producing ILs on a commercial scale.

Company	Product lines	Scale
Iolitec	Ammonium, imidazolium, phosphonium, piperidinium, pyridinium, pyrrolidinium, sulfonium ILs	Portfolio of >250 ILs [83] Multi-kg scale >1 tonne by 2020 [55]
Proionic	Imidazolium, pyrrolidinium ILs	>1 tonne
Scionix	Ammonium ILs [41,42,46]	Ten 200 kg batches of IL and one IL made on a tonne scale [46]
Solvionic	Ammonium, imidazolium, phosphonium, piperidinium, pyrrolidinium ILs	>1.5 tonnes/month by 2021 >50 tonnes/month by 2023 [189]

n.b. Several IL manufacturers that are often mentioned in the preceding literature are no longer operational (Bioniqs, Solvent Innovation GmbH) [190,191,192].
